# Determinants of depression among people with epilepsy in Central Ethiopia

**DOI:** 10.1186/s12991-018-0197-z

**Published:** 2018-06-13

**Authors:** Asrat Chaka, Tadesse Awoke, Zegeye Yohannis, Getinet Ayano, Minale Tareke, Andargie Abate, Mulugeta Nega

**Affiliations:** 1 Department of Psychiatry, Amanuel Mental Specialized Hospital, Addis Ababa, Ethiopia; 2 Department of Epidemiology and Biostatistics, University of Gondar, Gondar, Ethiopia; 3 College of Medicine and Health Science, Bahir Dar University, Bahir Dar, Ethiopia; 4 College of Medicine and Health Science, Haramaya University, Harer, Ethiopia

**Keywords:** Comorbidity, Depression, Epilepsy

## Abstract

**Background:**

Depression is the most frequently and highly occurring mental disorders in epilepsy patients. When depression is comorbid with epilepsy, it leads to low employment and poor quality of life. Thus, the aim of this study was to assess the prevalence and associated factors of depression among people living with epilepsy in Central Ethiopia.

**Methods:**

Institution-based cross-sectional study was conducted from April to May 2015 at Amanuel Mental Specialized and TikurAnbesa Hospitals, Addis Ababa, Ethiopia. Samples of 422 epilepsy patients were selected, and data on depression were collected using validated questionnaire using face-to-face interview technique. Logistic regression analysis was performed to assess predictors of depression.

**Results:**

The study indicated that the prevalence of depression among people with epilepsy was 43.8%. Factors associated with depression were being female (AOR 2.48; 95% CI, 1.61.3.81), being single (AOR 2.23; 95% CI 1.38–3.60), perceived stigma (AOR 2.47; 95% CI 1.59–3.83), medication adherence (AOR 2.85; 95% CI 1.64–4.96), and current substance use (AOR 2.10; 95% CI 1.34–3.30).

**Conclusion:**

There is a high prevalence of depression among epilepsy patients. Early detection and prompt management of depressive symptoms are critically important in reducing depression burden among people living with epilepsy.

## Introduction

Epilepsy is a disease of the brain defined by at least two unprovoked (or reflex) Seizures occurring within > 24 h apart; one unprovoked (or reflex) seizure and a probability of further seizures similar to the general recurrence risk (at least 60%) after two unprovoked seizures, occurring over the next 10 years [1]. It is a common neurological condition characterized by recurrent seizures and abnormal electrical activity in the brain that causes an involuntary change in body movement or function, sensation, awareness, or behavior [2]. More than 80% of people with epilepsy live in developing countries, and a majority of them do not have recourse to any effective treatment [3].

Depression is highly prevalent in the patient with epilepsy and is the most frequent comorbid psychiatric problem [4]. According to Diagnostic and Statistical Manual of Mental Disorders, Fifth Edition (DSM-5), depressive disorder is defined as at least one of the symptoms, either depressed mood or loss of interest or pleasure for at least 2 weeks, and includes at least other four symptoms from a list of criteria items, which are severe enough to cause severe distress or impairment in executing important functional roles [5].

Data from population-based studies have revealed a more complex, bidirectional relation between the two disorders, whereby not only are people with epilepsy at greater risk of developing depression but also people with depression are at greater risk of developing epilepsy. The existing common neurobiological pathogenic mechanisms shared by depressive disorders and epilepsy include neurotransmitter disturbances in the central nervous system such as serotonin, norepinephrine, dopamine, glutamate, and gamma-aminobutyric acid (GABA); endocrine disturbances such as hyperactive hypothalamic pituitary–adrenal axis, resulting in high serum concentrations of cortisol; and inflammatory mechanisms (in particular, interleukin-1β has been found to play a pathogenic role in patients with mood disorders) [6].

Depression is the most frequent psychiatric comorbidity in people with epilepsy with a prevalence rate ranging from 9.5 to 63% [7–12]. It leads to underemployment, lower rates of marriage, and a greater chance of social isolation when compared to counterparts [13, 14]. The high magnitude of depression among people living with epilepsy negatively influences their quality of life and increases suicidal tendency [15, 16]. Factors such as side effects of antiepileptic drugs, perceived stigma, fear of seizures, discrimination, joblessness, lack of social support, increased seizure frequency, and nonadherence to their medication have contributed to inducing depression among epilepsy patients [17, 18].

Despite this burden and consequences, there is a limited literature on the magnitude of depression and associated factors in people with epilepsy in the study area. Therefore, this study was intended to fill the gaps by assessing the prevalence and associated factors of depression among people living with epilepsy. It also helps to integrate mental health service in primary healthcare unit by early diagnosis and timely treatment of comorbid cases.

## Methods

### Study settings and population

Institution-based cross-sectional study was conducted from April to May 2015 at Amanuel Mental Specialized and TikurAnbesa Hospitals neurology clinics, located in Addis Ababa (capital city of Ethiopia). Amanuel Mental Specialized Hospital (AMSH) and TkurAnbesa Hospital (TAH) are the largest referral centers for people in Ethiopia as well as served as being medical teaching institutions. The AMSH and TAH have three and two neurologic outpatient clinics, respectively. Both hospitals provide medical and neurologic care.

The study populations comprised all epilepsy patients who were receiving treatments in their respective hospitals. All patients aged 18 years and above who have been diagnosed with epilepsy and provided with treatment in the outpatient epilepsy clinics in both hospitals were included in the study. Patients unable to communicate and seriously ill were excluded from the study.

### Sample size and Sampling procedures

The sample size was calculated using a single population proportion formula [*n*_o_ = (*Zα*_/2_)^2^ × (*P* − *q*))/*d*^2^, where *n*_o_ is the minimum sample size, *Z* the standardized normal distribution value at α/2, *P* the proportion of depression, and *d* is the margin of error]. By taking the proportion of depression at 49.3% [19], *Zα*_/2_ at 95% CI (1.96), and tolerable margin of error at (0.05), the minimum sample size was 384. By adding 10% for non-response rate, 423 participants were involved in the study.

Sampling interval was determined by dividing the total study population who had followed up during one month before data collection period (1600) by total sample size (422). Sampling fraction is 1600/422 ≈ 4. Hence, the sample interval is 4. The first study population was selected by lottery method, and the next study participants were chosen at regular intervals (every 4th), and the selected respondents were interviewed by data collectors.

### Data collection and quality assurance

Data were collected using instruments that measure depression, perceived stigma, Medication Adherence Scale, and social support-related questionnaires. The Patient Health Questionnaire (PHQ)-9 is the nine-item depression scale which is a powerful tool for clinicians to screen depression and monitor treatment response. The tool is based directly on the nine diagnostic criteria for major depressive disorder among epilepsy patients [20]. PHQ-9 is validated and extensively used in Ethiopia [21, 22]. The score of greater than or equal to 5 was considered to indicate probable depression in patients in this study. Patient medication adherence was measured using self-reported questions.

Perceived stigma was assessed via a three-item stigma scale with an overall possible score ranging from 0 (no felt stigma) to 3 (maximally felt stigma) [23]. Social support was measured by the three Oslo scale of social support measurement [24].

The questionnaire was designed and modified appropriately and translated into local language (Amharic) to be understood by all participants and translated back to English again to ensure its consistency. Training was given for four data collectors (psychiatry nurses) and one supervisor (BSc Nursing) for 2 days. The pretest was done at TikurAnbesa hospital 2 weeks before the day of actual data collection. The data collectors were supervised daily, and the filled questionnaires were checked properly by the supervisor and the principal investigator.

### Data management and processing

The coded data were checked, cleaned, and entered into epi.info version 3.5 and then exported into Statistical Package for the Social Sciences (SPSS) window version 20 for analysis. Descriptive statistic was used to explain the study participants in relation to study variable. Bivariate and multivariate logistic regression analyses were conducted to identify associated factors of depression among people with epilepsy. The strength of the association was interpreted by odds ratio with 95% CI, and the *p*-value less than 0.05 was considered as statistically significant.

### Ethical consideration

Ethical clearance was obtained after approval from the Institutional Review Board (IRB) of the College of Medicine and Health Sciences, the University of Gondar and from Amanuel Mental Specialized Hospital. The data collectors have clearly explained the aims of the study to the study participants. Information was collected after obtaining written consent from each participant. The right to exercise refusal was given to the study participants as well as their discontinuation from participation at any point in time. Confidentiality was maintained throughout the study. Those study participants suffering from recurrent severe suicidal thought was treated by communicating with psychiatry case team.

## Result

### Sociodemographic characteristics

The mean age of respondents was 31.57 (± SD 9.91). Nearly one-third (33.9%) of the respondents were between 25- and 34-aged groups and almost half of them were single (50. 2%) by marital status. A majority of them were living in the urban area (88.2%) (Table 1).

**Table 1:** Sociodemographic distribution of epilepsy patients on follow up at AMSH and TAH, 2015

Variables	Numbers	Percent (%)
Sex		
Male	249	59.0
Female	173	41
Age		
18–24	105	24.9
25–34	143	33.9
35–44	113	26.8
45–54	48	11.4
≥ 55	13	3.1
Ethnicity		
Amhara	138	32.7
Oromo	126	29.9
Tigre	24	5.7
Gurage	119	28.2
Others	15	3.6
Marital status		
Single	212	50.2
Divorce	58	13.7
Widowed	9	2.1
Married	143	33.9
Religion		
Orthodox	270	64.0
Muslim	98	23.0
Protestant	54	12.6
Residence		
Urban	372	88.2
Rural	50	11.8
Education		
Unable to read and write	24	5.7
Primary (1–8)	145	34.4
Secondary (9–12)	172	40.8
Diploma and degree	81	19.2
Occupation		
Not employed	41	9.7
Employed	381	90.3
With whom living now		
Family	396	93.8
Alone	14	3.3
Relative/friend	12	2.8

### Clinical- and medication-related characteristics

The majority (86%) of them had no family history of mental illness. Among the study respondents, nearly one-third (36%) had more than 10 years of duration of illness. Most (66.4%) of the study respondents were taking more than one antiepileptic drugs, had social support (85.3%), and had felt no stigma (62.3%) (Table 2).

**Table 2:** Description of clinical and psychosocial factors of patients with epilepsy at AMSH and TAH, 2015

Variables	Frequency	Numbers	Percent (%)
Family history of mental illness	Yes	59	14
	No	363	86
Seizure frequency per month	No seizure	243	57.6
	1–3	173	41.0
	4 and above	6	1.4
Duration of epilepsy illness	<1 year	19	4.5
	1–5 years	115	27.3
	6–10 years	136	32.2
	> 10 years	152	36
Types of epilepsy diagnosed	Grandmal	251	59.5
	Petimal	108	25.6
	Other	63	14.9
Type of antiepileptic drug	Mono therapy	142	33.6
	Polytherapy	280	66.4
Medication adherence	Adherence	330	78.2
	Poor adherence	92	21.8
Social support	Yes	360	85.3
	No	62	14.7
Perceived stigma	No felt stigma	263	62.3
	Felt stigma	159	37.7

### Substance related factors of the respondents

**Fig. 1:**
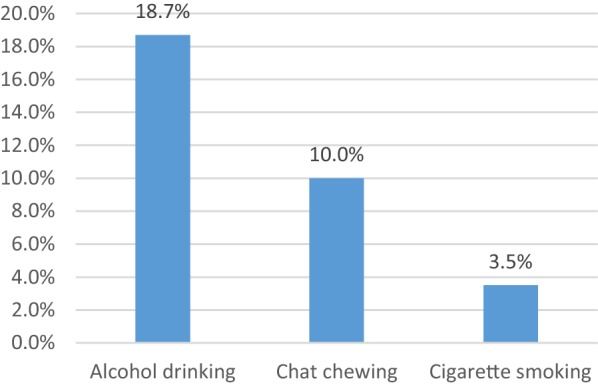
Bar graph showing the distribution of current substance use among epilepsy patients at AMSH and TAH, 2015

Most (67.8%) of the study participants had no history of substance use; however, the remaining (32.2%) used the substance. Among the total study participants who had used the substance, 79 (18.7%) were reported drinking alcohol (Fig. 1).

### Prevalence of depression among epilepsy patients

The distribution of PHQ-9 among respondents showed a mean score and standard deviation of 4.83 ± 4.68. Using cutoff point of five and above, 43.8% of the respondents had depression with 95% confidence interval (38.9%, 48. 8%). More females (52.4%) were affected by depression than males (47.6%) (Table 3).

**Table 3:** Factors associated with depression among epilepsy patients at under follow up at AMSH and TASH, 2015

Variables	Depression		COR (95% CI)	AOR (95% CI)
	Yes	No		
Sex				
Female	97	76	2.36 (1.56–3.47)	2.62 (1.68–4.09)*
Male	88	161	1.00	1.00
Marital status				
Single	110	102	2.35 (1.51–3.66)	2.04 (1.25–3.34)*
Divorced/separated	27	31	1.89 (1.01–3.54)	
Widowed	3	6	1.089 (0.26–4.55)	
Married	45	98	1.00	1.00
Medication adherence				
Yes	162	168	1.00	1.00
No	23	69	2.89 (1.72–4.86)	2.65 (1.52–4.65)*
Perceived stigma				
No	94	169	1.00	1.00
Yes	91	68	2.40 (1.60–3.60)	2.65 (1.65–4.07)*
Current substance use (alcohol, chat, cigarette)				
Yes	77	59	2.15 (1.42–3.26)	2.14 (1.34–3.39)*
No	108	178	1.00	1.00

* p < 0.05; 1.00 = Reference; *COR* crude odds ratio, *AOR* adjusted odds ratio

### Factors associated with depression among epilepsy patients

The associations of all potential explanatory variables and depression were checked using logistic regression model. However, only being female, single, perceived stigma, poor medication adherence, and current use of substance were significantly associated with depression in bivariate and multivariate logistic regression (*p* < 0.05).

Being female was 2.6 times more likely to have depression when compared to males (AOR 2.62, 95% CI 1.68–4.09). Similarly, being single was around twice more likely to have depression compared to married participants (AOR 2.04, 95% CI 1.25–3.34).

Moreover, those who have poor medication adherence and having perceived stigma had close to three odds of having depression compared to their counterparts (AOR 2.65, 95% CI 1.52–4.65; AOR 2.65, 95% CI 1.65–4.07, respectively). In addition, current substance use (alcohol drinking, Khat chewing, and cigarette smoking) were significantly associated with depression. Depression was two times more likely among current substance users (AOR 2.14, 95% CI 1.34–3.39) (Table 3).

## Discussion

In this study, the prevalence rate of depression among epilepsy patients was 43.8% (38.9, 48.8%). It is consistent with the study done at the University of Gondar Hospital (45.2%) [25] and Sudan (45.5%) [26].

This finding was lower than the result from the study conducted at the Jimma University Specialized Hospital (49.3%) [19]. Other studies conducted in Nigeria and Gaza stated that depression had been prevalent among 85.5 and 63% of participants, respectively [9, 27] which are very high compared with the current study, and they were almost three times higher in magnitude than that reported in a study done in Canada [11]. This difference might be due to the sociocultural variation and instrument, since those authors used BDI which has items similar to somatic complaints, and this might have led to overestimating the prevalence.

However, the current study’s finding regarding prevalence rate is higher than the report described in Amanuel Mental Specialized Hospital (33.3%) [28], Kenya (16.5%) [29], Iran (9.5%) [8], Thailand (20%) [30], and Greek (22.5%) [31]. These discrepancies might be due to the difference in study participants, method, culture, time, and settings.

In this study, the researchers found a high prevalence of depression among female compared to male respondents which are consistent with the study done in Gaza [9]. However, the study in Nigeria and Ethiopia [19, 27] revealed that no significant relationship between gender and depression in people with epilepsy. The difference is most likely due to diverse methodological approaches, cultural variation, and different instruments they used to measure depression. In general, females faced difficulty in performing normal activities of daily living, and they might face several risks or hardships with regard to reproductive activity and pregnancy. Furthermore, women with epilepsy face difficulty in decision-making with regard to important major life events such as marriage or bearing children. Thus, these consequences might increase depression among females.

With regard to marital status in this study, those who were married were less likely to be depressed compared to those with single status, and having another marital status. This finding was consistent with the previous study in Jimma Ethiopia [19] but inconsistent with other studies in which the authors found no significant difference among different marital statuses [27, 28]. This might be due to marriage-related change of lifestyles because of some kind of guardian-like protective effect of the marital status where adherence to healthy activities is greatly increased—and behaviors leading to health risks are reduced. Moreover, married people also have higher levels of emotional support.

The study has also shown that patients who were not adherent to their medication had depression compared to those who were adherent. This hypothesis is supported by previous studies [19, 25]. The high rates of poor adherence demonstrated in this study are causing concerns, given the consequences of antiepileptic discontinuation. It might be assumed that patients who discontinue medication will be more likely to relapse and have very poor and less control over the disease than those who continue medications.

Moreover, in this study, stigma was associated with depression which is supported by different studies [19, 28]. The previous study conducted in Ethiopia among epilepsy among patients found perceived stigma to be a common problem among people living with epilepsy [32]. This is because people with epilepsy might be overprotected and restricted from doing many activities by their family members, friends, or teachers. Overprotection arising from stigmatization can have severe consequences. Eventually, this stigma shatters a person’s hope and self-esteem leading to negative outcomes related to recovery including depressive symptoms, social avoidance, and a preference for adopting avoidance of coping strategies.

In this particular study, current substance use has a significant association with depression. This result is supported by Epilepsy Action Australia report [33]. Substance use among people taking antiepileptic medications is likely to be more sensitive to the effects of substances. The substance can interfere with the metabolism of these medications and therefore increase the chance of seizures. Some medications can enhance the toxic effects of alcohol, and people can feel severely intoxicated after drinking only a small amount. Skipping a dose, taking extra medication, or altering the time of taking regular antiepileptic medications before drinking will not alter this reaction but may cause additional adverse effects or seizures [33].

In general, the implication of this finding indicates that people living with epilepsy need strong counseling in terms of adherence, by way of creating awareness in the community and addressing misperception issues attached to epilepsy. Collaborative efforts among different stakeholders and clinicians are recommended to bring effective management strategies to neurologic clinics. In addition, generating additional evidence through further research is required.

A limitation of this study was that it was not possible to clarify the cause–effect relationship between depression and epilepsy due to the cross-sectional nature of the study. A prospective study could help to establish clearly whether epilepsy predisposes to depression or a consequence of depression predispose to epilepsy. Another limitation includes recall bias—regarding the duration of illness and substance-use-related factor—which was not assessed by a standard tool.

## Conclusion

The finding showed that there is a high prevalence of comorbid depression (43.8%) among epilepsy patients at the AMSH and TAH. The epilepsy-related sociodemographic variables like being female, single, and clinical-related factors including poor medication adherence, current substance use, and perceived stigma were significantly associated with depression. Early recognition of depression symptoms in people with epilepsy should be of great concern for healthcare providers to help them provide appropriate counseling regarding adherence and substance use.
